# Supplementary material for: Differences in the Emission of Volatile Organic Compounds (VOCs) between Non-Differentiating and Adipogenically Differentiating Mesenchymal Stromal/Stem Cells from Human Adipose Tissue
Source: Cells. 2019 Jul 10;8(7):697. doi: 10.3390/cells8070697 (PMC6678290; doi:10.3390/cells8070697)
Supplement: Supplementary file 1 [file cells-08-00697-s001.pdf]

cells

Supplements

Table S1 Quantitative parameters of VOCs: Concentration ranges, limits of detection (LOD), limits of quantification (LOQ) and standard deviation (SD) of 11 substances detected in ASC and pure culture medium, respectively.

Substance	Concentration range in ASCs	SD [% at nmol/L]	Limit of detection [nmol/L]	Limit of quantification [nmol/L]
**Acetaldehyde**	<LOQ – 953.36	9 % (at 4.1 nmol/L)	0.63	0.95
**Pentane**	<LOQ. – 1.13	13 % (at 0.4 nmol/L)	0.15	0.22
**1.3-Di-tert-butylbenzene**	<LOQ – 0.13	28 % (at 0.4 nmol/L)	0.0006	0.0007
**Ethylbenzene**	<LOQ. – 0.04	2 % (at 0.4 nmol/L)	0.0008	0.014
**Heptanal**	0.042 – 0.28	4 % (at 0.6 nmol/L)	0.023	0.038
**Benzaldehyde**	<LOQ – 0.15	12 % (at 0.4 nmol/L)	0.007	0.01
**Octanal**	0.05 –0.27	6% (at 0.6 nmol/L)	0.03	0.04
**Acetone**	0.66 – 3.40	9 % (at 0.6 nmol/L)	0.14	0.2
**Tert-Butanol**	0.03 – 0.60	9 % (at 0.4 nmol/L)	0.005	0.007
**2-Butanone**	0.77 – 3.45	6 % (at 0.6 nmol/L)	0.02	0.03
**2-Ethylhexanol**	<LOD. – 0.78	17 % (at 0.4 nmol/L)	0.008	0.01

Table S2: P-values of statistical comparison between non- differentiating ASC (non-diff ASC) and adipogenically differentiating (adipo ASC). Statistical analysis was done by Kruskal-Wallis rank sum test and pairwise comparisons using Tukey and Kramer (Nemenyi) test. Non-significant P-values are stated with **n.s**. and significant P-values (<0.05) are stated with **<0.05**.

**Acetaldehyde**		**Non-diff ASC**				**Adipo ASC**		
		**Day 1**	**Day 7**	**Day 14**	**Day 21**	**Day 1**	**Day 7**	**Day 14**
**Non-diff ASC**	**Day 7**	n.s.	-	-	-	-	-	-
	**Day 14**	n.s.	n.s.	-	-	-	-	-
	**Day 21**	n.s.	n.s.	n.s.	-	-	-	-
**Adipo ASC**	**Day 1**	n.s.	n.s.	n.s.	n.s.	-	-	-
	**Day 7**	n.s.	<0.05	<0.05	n.s.	n.s.	-	-
	**Day 14**	n.s.	<0.05	<0.05	<0.05	n.s.	n.s.	-
	**Day 21**	n.s.	<0.05	<0.05	<0.05	n.s.	n.s.	n.s.

**Pentane**		**Non-diff ASC**				**Adipo ASC**		
		**Day 1**	**Day 7**	**Day 14**	**Day 21**	**Day 1**	**Day 7**	**Day 14**
**Non-diff ASC**	**Day 7**	n.s.	-	-	-	-	-	-
	**Day 14**	n.s.	n.s.	-	-	-	-	-
	**Day 21**	n.s.	n.s.	n.s.	-	-	-	-
**Adipo ASC**	**Day 1**	n.s.	n.s.	n.s.	n.s.	-	-	-
	**Day 7**	n.s.	<0.05	n.s.	n.s.	<0.05	-	-
	**Day 14**	n.s.	<0.05	n.s.	n.s.	<0.05	n.s.	-
	**Day 21**	n.s.	n.s.	n.s.	n.s.	n.s.	n.s.	n.s.

**1.3 bis (1.1 dimethylethyl) Benzene**		**Non-diff ASC**				**Adipo ASC**		
		**Day 1**	**Day 7**	**Day 14**	**Day 21**	**Day 1**	**Day 7**	**Day 14**
**Non-diff ASC**	**Day 7**	n.s.	-	-	-	-	-	-
	**Day 14**	n.s.	n.s.	-	-	-	-	-
	**Day 21**	n.s.	n.s.	n.s.	-	-	-	-
**Adipo ASC**	**Day 1**	n.s.	n.s.	n.s.	n.s.	-	-	-
	**Day 7**	n.s.	n.s.	n.s.	n.s.	n.s.	-	-
	**Day 14**	n.s.	n.s.	n.s.	n.s.	n.s.	n.s.	-
	**Day 21**	n.s.	<0.05	n.s.	<0.05	n.s.	n.s.	n.s.

**Ethylbenzene**		**Non-diff ASC**				**Adipo ASC**		
		**Day 1**	**Day 7**	**Day 14**	**Day 21**	**Day 1**	**Day 7**	**Day 14**
**Non-diff ASC**	**Day 7**	n.s.	-	-	-	-	-	-
	**Day 14**	n.s.	n.s.	-	-	-	-	-
	**Day 21**	n.s.	n.s.	n.s.	-	-	-	-

**Adipo ASC**	**Day 1**	n.s.	n.s.	n.s.	n.s.	-	-	-
	**Day 7**	n.s.	n.s.	n.s.	n.s.	n.s.	-	-
	**Day 14**	n.s.	n.s.	n.s.	<0.05	n.s.	n.s.	-
	**Day 21**	n.s.	n.s.	n.s.	<0.05	n.s.	n.s.	<0.05.

**Benzaldehyde**		**Non-diff ASC**				**Adipo ASC**		
		**Day 1**	**Day 7**	**Day 14**	**Day 21**	**Day 1**	**Day 7**	**Day 14**
**Non-diff ASC**	**Day 7**	n.s.	-	-	-	-	-	-
	**Day 14**	n.s.	n.s.	-	-	-	-	-
	**Day 21**	n.s.	n.s.	n.s.	-	-	-	-
**Adipo ASC**	**Day 1**	<0.05	n.s.	n.s.	n.s.	-	-	-
	**Day 7**	<0.05	n.s.	<0.05	<0.05	n.s.	-	-
	**Day 14**	<0.05	n.s.	<0.05	<0.05	n.s.	n.s.	-
	**Day 21**	<0.05.	n.s.	n.s.	n.s.	n.s.	n.s.	n.s.

**Heptanal**		**Non-diff ASC**				**Adipo ASC**		
		**Day 1**	**Day 7**	**Day 14**	**Day 21**	**Day 1**	**Day 7**	**Day 14**
**Non-diff ASC**	**Day 7**	n.s.	-	-	-	-	-	-
	**Day 14**	n.s.	n.s.	-	-	-	-	-
	**Day 21**	<0.05	n.s.	n.s.	-	-	-	-
**Adipo ASC**	**Day 1**	n.s.	n.s.	n.s.	n.s.	-	-	-
	**Day 7**	<0.05.	n.s.	n.s.	n.s.	n.s.	-	-
	**Day 14**	n.s.	n.s.	n.s.	n.s.	n.s.	n.s.	-
	**Day 21**	n.s.	n.s.	n.s.	n.s.	n.s.	n.s.	n.s.

VOCs from adipogenically differentiating ASC

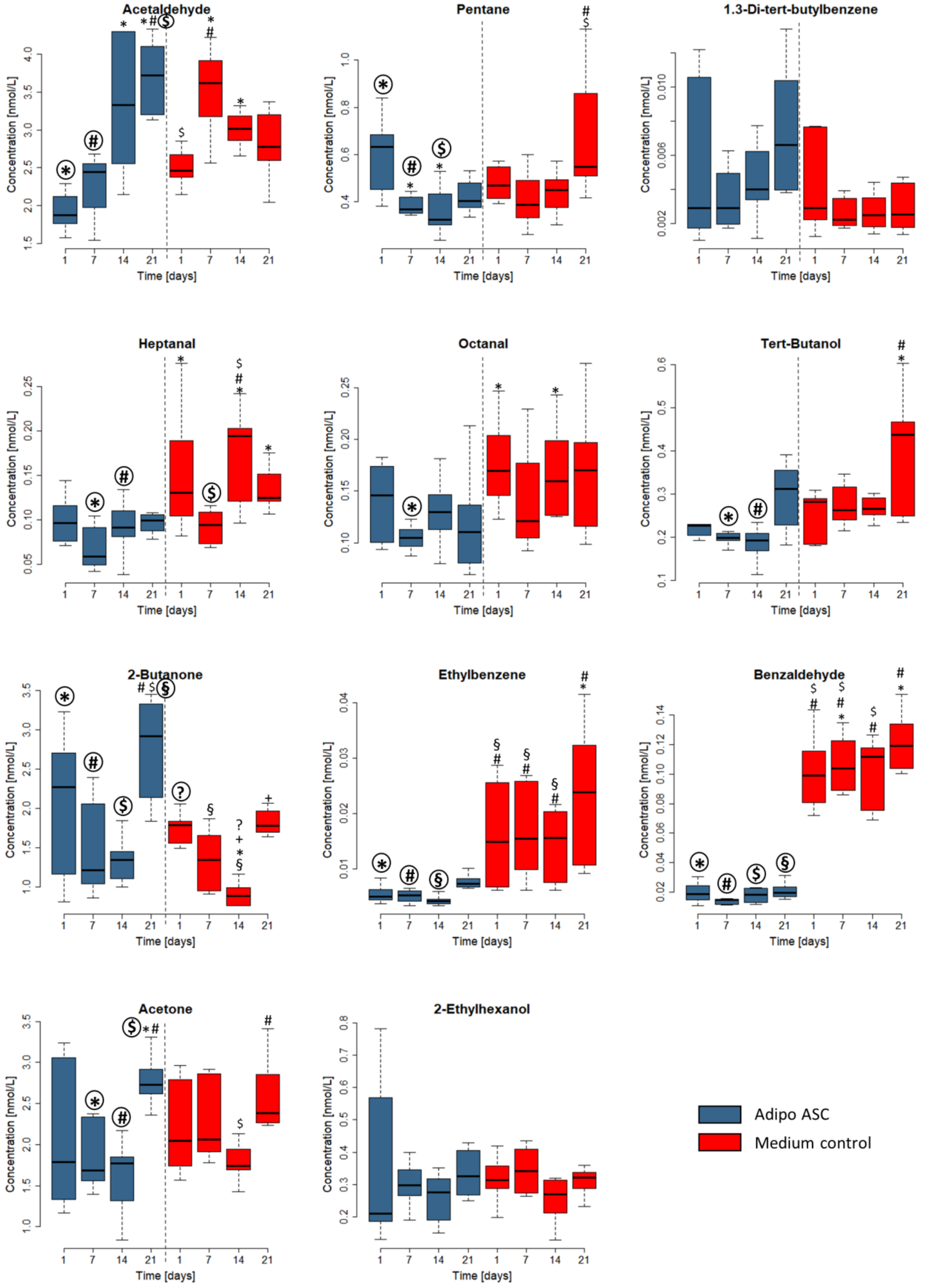

Figure S1 VOCs during the adipogenic differentiation. Concentrations are shown on the y axis in nmol/L. The x-axis shows the time point of measurements. Cell cultures with adipogenically differentiating ASC (Adipo ASC, blue), media samples without cells (medium control, red). Boxplots represent data of three independent differentiation experiments. Significance was tested within all groups. Symbols (*, #, $, §, +, ?) indicate significant differences to the corresponding highlighted group (p-values < 0.05).

VOCs from non-differentiating ASC

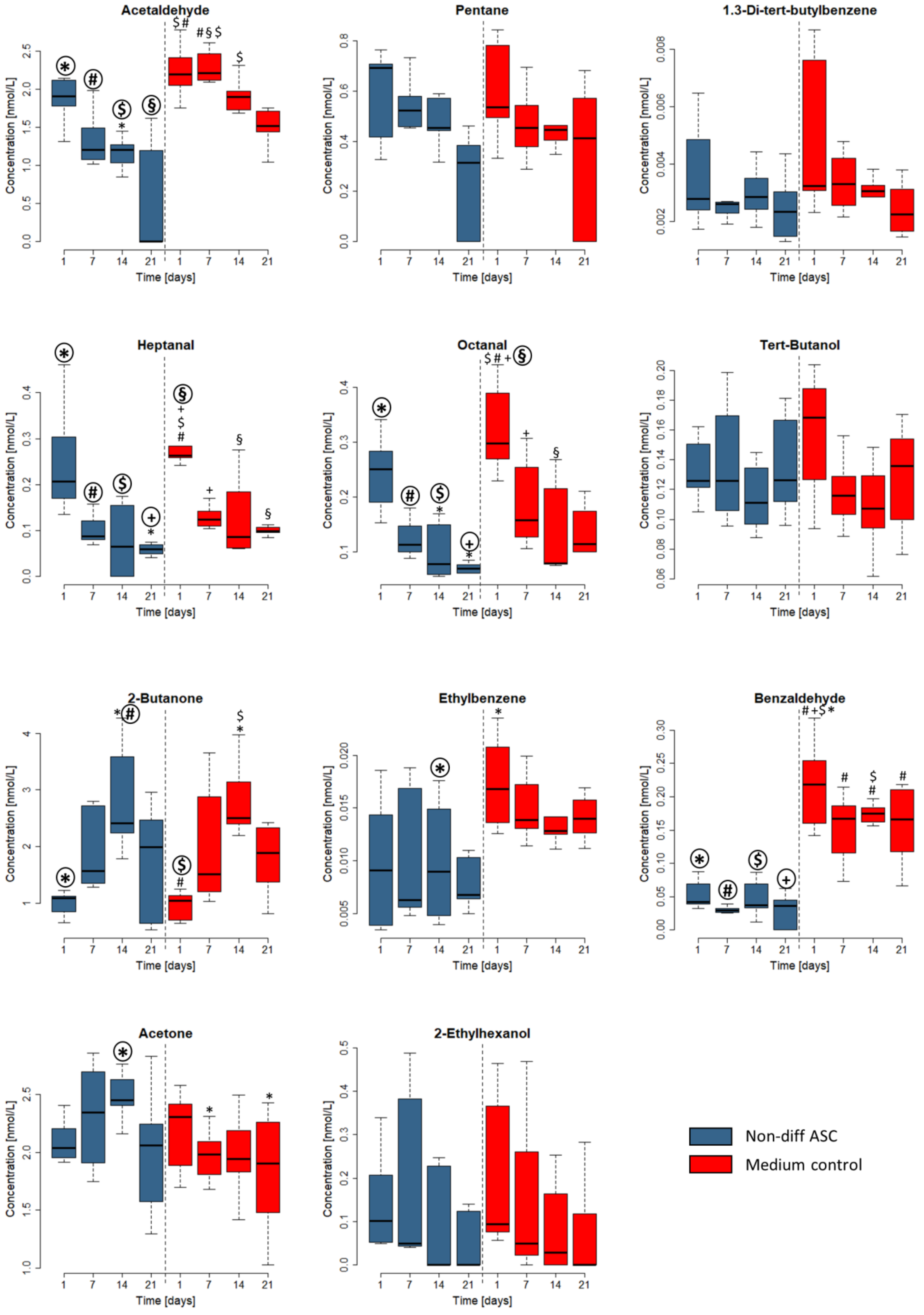

Figure S2 VOCs of non-differentiating ASC. The concentration is shown on the y axis in nmol/L. The x-axis shows the measurement time point. Red boxplots indicate the data from pure medium samples and blue box plots indicate the data from the cell culture. The boxplots represent the data of three independent differentiation experiments. Significance was tested within all groups. Symbols (*, #, $,+, §) indicate significant differences to the corresponding highlighted group (p-values < 0.05).
